# Excess Risk of Maternal Death from Sickle Cell Disease in Jamaica: 1998–2007

**DOI:** 10.1371/journal.pone.0026281

**Published:** 2011-10-24

**Authors:** Monika R. Asnani, Affette M. McCaw-Binns, Marvin E. Reid

**Affiliations:** 1 Sickle Cell Unit, Tropical Medicine Research Institute, University of the West Indies, Mona, Kingston, Jamaica; 2 Department of Community Health and Psychiatry, University of the West Indies, Mona, Kingston, Jamaica; University of Sao Paulo – USP, Brazil

## Abstract

**Background:**

Decreases in direct maternal deaths in Jamaica have been negated by growing indirect deaths. With sickle cell disease (SCD) a consistent underlying cause, we describe the epidemiology of maternal deaths in this population.

**Methods:**

Demographic, service delivery and cause specific mortality rates were compared among women with (n = 42) and without SCD (n = 376), and between SCD women who died in 1998–2002 and 2003–7.

**Results:**

Women with SCD had fewer viable pregnancies (p: 0.02) despite greater access to high risk antenatal care (p: 0.001), and more often died in an intensive care unit (p: 0.002). In the most recent period (2003–7) SCD women achieved more pregnancies (median 2 vs. 3; p: 0.009), made more antenatal visits (mean 3.3 vs. 7.3; p: 0.01) and were more often admitted antenatally (p:<0.0001). The maternal mortality ratio for SCD decedents was 7–11 times higher than the general population, with 41% of deaths attributable to their disorder. Cause specific mortality was higher for cardiovascular complications, gestational hypertension and haemorrhage. Respiratory failure was the leading immediate cause of death.

**Conclusions:**

Women with SCD experience a significant excess risk of dying in pregnancy and childbirth [MMR: (SCD) 719/100,000, (non SCD) 78/100,000]. MDG5 cannot be realised without improving care for women with SCD. Tertiary services (e.g. ventilator support) are needed at regional centres to improve outcomes in this and other high risk populations. Universal SCD screening in pregnancy in populations of African and Mediterranean descent is needed as are guidelines for managing SCD pregnancies and educating families with SCD.

## Introduction

**Figure 1:**
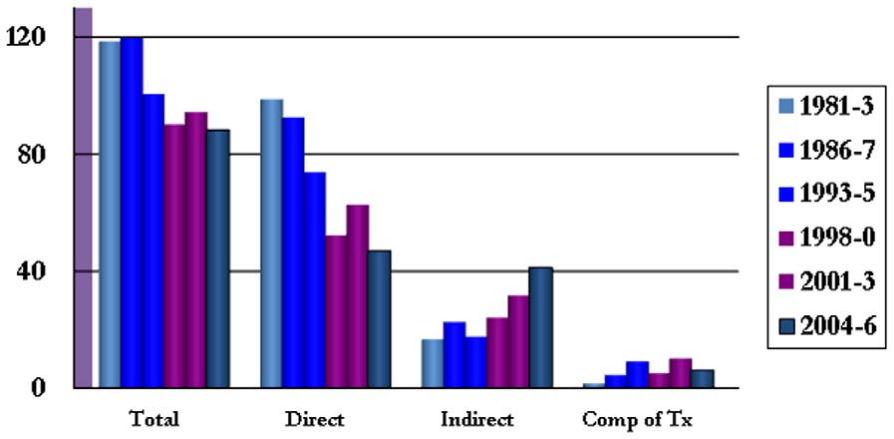
Maternal mortality ratios (per 100 000 live births) in Jamaica, 1981–2006.

While maternal mortality has declined in Jamaica,[1] due mainly to efforts aimed at reducing direct deaths, the contribution from indirect deaths has risen from 17% in 1993–1995 to 31% in 2001–2003 (Figure 1), with sickle cell disease (SCD) a consistent associated condition or underlying cause.

SCD is the commonest genetic disorder in Jamaica, with 10% of the population carrying the S sickle gene. Among live born infants, 0.33% are homozygous SCD (SS) and 0.67% have some form of SCD.[2] The disease is associated with high lifetime rates of morbidity and premature mortality. Complications of SCD affect every organ system and can be aggravated by pregnancy.[3] Better quality of care for persons with SCD has improved survival and thus the number of women entering the reproductive age group. Reproduction however, is less efficient in this population. Sergeant et al reported lower fecundity among women with SS compared to those with heterozygous SCD (SC) or the normal genotype (AA), characterized by later median age at menarche and first pregnancy, more spontaneous abortions, fewer live births and lower mean birth weight and gestational age at delivery among those able to conceive. Successful pregnancy outcomes were reported for only 57% of SS women compared to 85% with SC and 89% of AA controls. [4]

International experience with pregnancy outcomes in SCD has been varied. In Bahrain, Rajab et al[5] reported that maternal morbidity and mortality and perinatal losses are a significant consideration in SCD pregnancies. El-Shafei et al declared them as high-risk pregnancies requiring close specialist monitoring and reported SCD to be the underlying cause of death in 12 of 37 (32%) maternal deaths.[6]

Among antenatal women of African descent in Massachusetts, USA, the prevalence of SCD was 0.6%. They had higher rates of fetal death: OR 2.2 (95% CI = 1.2, 4.2), preterm delivery; 1.5 (95% CI = 1.2, 1.8), low birth weight 1.7 (95% CI = 1.1, 2.6), and small for gestational age (SGA) babies 1.3 (95% CI = 1.0, 1.7).[7] The Cooperative Study of SCD has shown favourable outcomes, with antepartum and intrapartum complication rates similar to pregnancies in African-American women without SCD[8] however 21% of women had SGA babies. Sun et al from Grady Hospital in Georgia, USA [9] also reported favourable pregnancy outcomes, however women experienced more intrauterine growth retardation, antepartum hospitalizations, postpartum infections and prematurity compared to those with the normal AA genotype. None of these series showed any significant maternal mortality.

Women with SCD had similar levels of obstetric complications (e.g. pre-eclampsia, urinary tract infection) as AA counterparts in Nigeria, West Africa, a population with similar genetic roots as ours.[10] Studies from the United States however suggest that women with SCD had higher odds of complications such as the hypertensive disorders of pregnancy, including pre-eclampsia[11] stroke,[12] and venous thromboembolism.[13] Among severely ill women admitted with multiple organ failure to a Havana hospital between 1998 and 2006, half of whom died, sickle cell disease was the leading medical complication.[14]

While the Sickle Cell Unit (SCU), at the University Hospital of the West Indies has been a centre of research, service and outreach for persons living with this genetic disorder in Jamaica, most women with SCD access the same antenatal services available to women without SCD, especially residents outside the Kingston Metropolitan area. This study aims to describe the epidemiology of maternal deaths among women with SCD over a decade across the island who access care through the general antenatal services, some of whom may be referred to the SCU for consultation, to better understand the needs of this population. The objectives of the study are to:

- Describe the demographic characteristics (age, parity, residence) and obstetric experience (#pregnancies, #live births, complications) of SCD women with pregnancy related deaths and compare these characteristics with non SCD women who also died and across two 5 year periods (1998–2002; 2003–2007)
- Determine the immediate and underlying causes of deaths among women with and without SCD and whether the causes have changed over the two 5 year periods of observation
- Identify access to health service factors which may be associated with risk of dying.

## Methods

### Ethics Statement

The study was granted ethical approval by the University of the West Indies/ University Hospital of the West Indies Ethical Committee. The study was designed and performed in adherence with the Declaration of Helsinki.

Maternal deaths are a Class 1 Notifiable event to Ministry of Health, Jamaica and as such all maternal deaths are recorded by the Ministry. As such, no consent was needed to study this condition and access to these data was provided by the Directors of Family Health Services and Epidemiology, Ministry of Health, Jamaica.

### Procedures

The study employs data from the Jamaican maternal mortality surveillance system, instituted in 1998. [15] An active surveillance system, suspected maternal deaths are reported to the Ministry of Health, and investigated by the local health team. A 2004 evaluation documented that from 1998 to 2003 increasing number of cases were being reported, however first trimester deaths and late maternal deaths, especially occurring on non-obstetric wards and in the Accident and Emergency departments were more likely to be missed.[15] These findings were used to improve the surveillance system by explicitly including the reporting of late maternal deaths, instituting surveillance in non-obstetric areas of public hospitals, private facilities and in the community. Over 95% of deliveries occur in public facilities.[16] Maternal deaths reported from public hospitals between 1998 and 2007 were independently validated by reviewing all hospital deaths in women 10–50 years to determine whether they were pregnancy related to ensure that all such events were included in the database.

**Figure 2:**
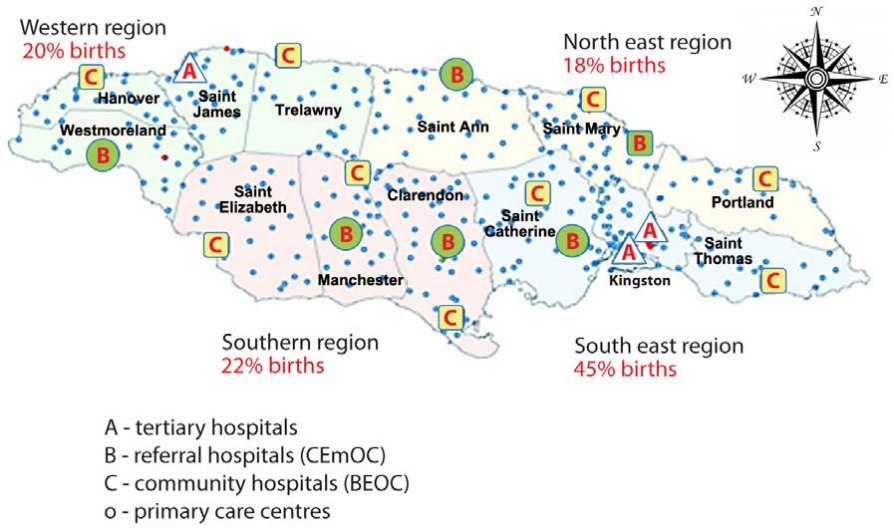
Jamaica: Hospitals and health centres.

We examined whether there were qualitative differences in access to care by health regions (south-east, northeast, west, and south). Given the highly specialized need for care in this population, we also examined whether there were mortality differences according to the highest level of care (access to care) in the women's parish of residence. This is defined as tertiary care, where hospitals provide intensive care (ICU), including ventilator support, limited to three parishes: Kingston, St Andrew and St James; comprehensive obstetric (referral) care, with obstetric and paediatric consultants based at regional hospitals (St Mary, St Ann, Westmoreland, Manchester, St Catherine); and midwifery care only, where delivery services in the parish are provided by midwives, supported by a surgeon for emergency Caesarean section (remaining five parishes: St Thomas, Portland, Trelawny, Hanover, St Elizabeth). Complicated cases from these parishes should be transferred to the nearest regional or tertiary hospital (See Figure 2).

The causes of death and complications were coded using ICD 10. Underlying causes were combined into major groupings and classified as direct, indirect or due to complications of treatment.[17] The immediate cause of death was summarized according to organ system dysfunction criteria.[18]

To determine how many antenatal women had SCD, we utilized Ministry of Health data on the prevalence of SCD in their antenatal population. Since 2001 (Appendix S1), women are screened for anaemia and the sickling disorder; among those failing the screen, electrophoresis is done to determine their genotype. From 2001–7, 1.57% of first antenatal visits or 1.0% of registered live births were positive for HbSS or HbSC. Applying these prevalence rates to births over the decade leads to an estimated 4863 to 7617 women with SCD who gave birth over the period. Maternal mortality ratios (deaths/100 000 live births) were computed using both estimates of the SCD population.

### Statistics

A ‘Reproductive Index’ was calculated to determine the proportion of pregnancies reaching viability (Parity/Gravidity). Descriptive statistics are reported as frequency and percent for categorical data and compared using χ^2^ test or Fisher's exact test, as appropriate. Continuous data are reported as mean and standard deviation (SD) or median and interquartile range (IQR), and compared by Student's *t* test. Data were analysed using STATA™ 10.0 (StataCorp, College Station, Texas, USA).

## Results

**Figure 3:**
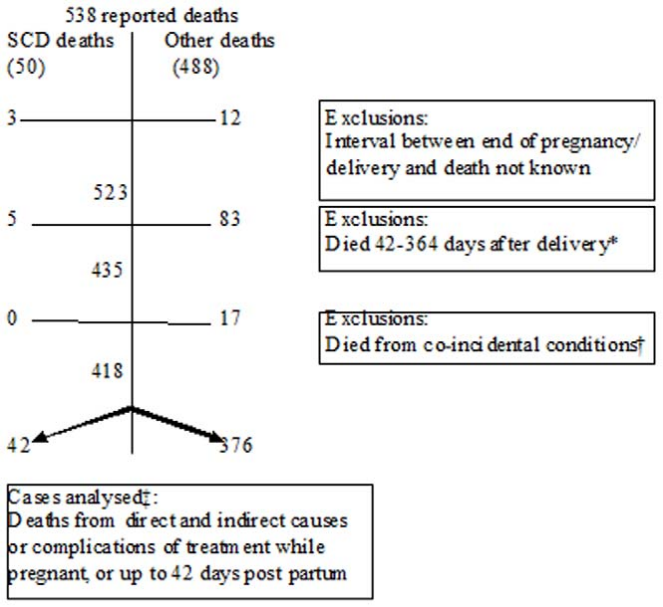
Case selection process.

Of 538 pregnancy related deaths identified from 1998–2007, 50 (9.3%) occurred in women with SCD. Consistent with the WHO definition of maternal death, exclusions included 15 women whose interval between delivery and death was unknown (three SCD, 12 non SCD); 88 late deaths (five SCD, 83 non SCD) and 17 non SCD coincidental deaths, yielding a study population of 42 (SCD) and 376 non SCD cases for analysis (Figure 3).

### SCD deaths

The mean age of SCD women was 27.5 years (range: 17–37 years). The genotype was SS in 40 and SC in the remaining 2 women. Median parity was 1 (range 0–4) and gravidity was 3 (range 1–5). Most deaths, (n = 28, 67%) occurred in the south-east region where 45% of births occur, with almost equal representation from the other three regions responsible for 10% (north-east), 10% (west) and 14% (south) of births. Most deaths (n = 38; 90%) occurred in hospital.

### SCD versus non-SCD maternal deaths

Table 1 compares maternal deaths in women with and without SCD. While there were no differences in the mean age nor the number of pregnancies, women with SCD had significantly lower parity, i.e. their reproductive index was significantly lower than women without SCD. Those with SCD were more likely to be enrolled in high risk antenatal care facilities, made almost twice as many antenatal visits and had greater access to tertiary level care. Significantly more SCD maternal deaths occurred in intensive care facilities. There were however no differences in gestational age at delivery or outcome of pregnancy.

**Table 1:** Socio-demographics, access to care, health service utilization and pregnancy outcome characteristics of maternal deaths, Jamaica: 1998–2007, by sickle cell disease (SCD) status.

Variable	All other maternal deaths (n = 376)	SCD maternal deaths (n = 42)	p-value
**Socio-demographic characteristics**			
Age in years (mean ± SD)	29.4±7.2	27.5±5.6	0.103
Gravidity, Median (IQR)	3 (2–5)	3 (2–3)	0.2
Parity, Median (IQR)	2 (0–3)	1 (0–2)	**0**.**01**
Reproductive Index* (mean±SD)	0.44±0.3	0.31±0.3	**0**.**02**
**Access to care**			
Region of residence, n (%)			0.083
Southeast	171 (45.7)	28 (66.7)	
Northeast	53 (14.2)	4 (9.5)	
West	58 (15.5)	4 (9.5)	
South	92 (24.6)	6 (14.3)	
Highest level hospital facility in parish of residence, n (%)			**0**.**01**
Tertiary care	122 (33.1)	24 (57.1)	
Referral obstetric care	121 (32.8)	11 (26.2)	
Midwifery care	126 (34.1)	7 (16.7)	
Antenatal care, n (%)			**0**.**001**
None reported	61 (16.2)	5 (11.9)	
Primary health care	105 (27.9)	6 (14.3)	
Hospital, high risk	82 (21.8)	22 (52.4)	
Private doctor	53 (14.1)	5 (11.9)	
Not known	75 (19.9)	4 (9.5)	
Number of antenatal visits, (mean±SD)	3.27±3.33	6.03±4.42	**<0**.**0001**
Attendant at delivery, n (%)			0.522
Traditional birth attendant/self	14 (4.8)	1 (2.9)	
Registered midwife	63 (21.6)	4 (11.8)	
Obstetrician	107 (36.6)	15 (44.1)	
Other medical practitioner	108 (37.0)	14 (41.2)	
Place of death, n (%)			**0**.**002**
Intensive care unit	41 (11.0)	14 (33.3)	
Type a	147 (39.5)	14 (33.3)	
Type b	92 (24.7)	6 (14.3)	
Type c	62 (16.7)	4 (9.5)	
Other	30 (8.1)	4 (9.5)	
**Pregnancy outcome**			
Days to death, n (%)			0.656
Pregnancy- 42 days post-partum			
Undelivered	104 (28.7)	10 (24.4)	
<24 hours	70 (19.3)	9 (22.0)	
1–6 Days	97 (26.7)	14 (34.1)	
7–42 Days	92 (25.3)	8 (19.5)	
Outcomes of pregnancy, n (%)			0.512
Died undelivered	98 (27.4)	8 (19.5)	
Early foetal loss	57 (15.9)	5 (12.2)	
Stillbirth	52 (14.5)	6 (14.6)	
Live birth	151 (42.2)	22 (53.7)	
Survival of infant, n (%)			0.234
Yes	121 (32.2)	19 (45.3)	
No	219 (58.2)	20 (47.6)	
Not known	36 (9.6)	3 (7.1)	
Gestational age in weeks at termination of pregnancy/death (mean±SD)	29.9±0.1(n = 302)	32.4±.7(n = 36)	0.15
Trimester pregnancy ended/death			0.268
First (0–14 weeks)	57 (16.6)	3 (7.3)	
Second (15–28 weeks)	70 (20.4)	8 (19.5)	
Third (29+ weeks)	216 (63.0)	30 (73.2)	
Birth Weight of live born infants, kgs(mean±SD)	2.55±98(n = 116)	2.62±0.94(n = 19)	0.74

*Calculated as Parity/ Gravidity: Defined as the proportion of pregnancies reaching viability.

### SCD related maternal deaths during 1998–2002 versus 2003–2007

Women in the second study period tended to be older but not significantly (Table 2); and achieved more successful pregnancies than earlier (p = 0.009). They had higher enrolment at high risk antenatal care facilities (which were being developed and expanded during this time period), made more antenatal visits, had more antepartum hospitalizations and were more often delivered by obstetricians. No differences were seen in gestational age at delivery, place of delivery or death (although more died in an ICU), or infant survival nor in the immediate or underlying causes of death.

**Table 2:** Characteristics of SCD related maternal deaths in 1998–2002 and 2003–2007.

Variable	1998–2002(n = 16)	2003–2007(n = 26)	p-value
**Socio-demographic characteristics**			
Age in years (mean±SD)	25.9±7.1	28.5±4.4	0.197
Gravidity, Median (IQR)	2 (1–3)	3 (3–4)	**0**.**009**
Parity, Median (IQR)	0 (0–1)	1 (0–2)	0.25
Reproductive Index* (mean±SD)	0.25±0.29	0.34±0.29	0.375
**Access to care**			
Region of residence, n (%)			0.821(Fishers)
Southeast	11 (68.8)	17 (65.4)	
Northeast	1 (6.2)	3 (11.5)	
West	1 (6.2)	3 (11.5)	
South	3 (18.8)	3 (11.5)	
Highest level hospital facility in parish of residence, n (%)			0.680 (Fishers)
Type a (tertiary care)	10 (62.5)	14 (53.8)	
Type b (referral obstetric care)	3 (18.8)	8 (30.8)	
Type c (midwifery care)	3 (18.8)	4 (15.4)	
Antenatal care, n (%)			**0**.**008 (Fishers)**
None reported	2 (12.5)	3 (11.5)	
Primary health care	4 (25.0)	3 (11.5)	
Hospital, high risk	4 (25.0)	18 (69.2)	
Private doctor	2 (12.5)	3 (11.5)	
Not known	4 (25.0)	0 (0.0)	
Number of antenatal visits, (mean±SD)	3.27±2.8	7.29±4.5	**0.01**
Ante partum admission, n (%)			**<0.0001(Fishers)**
Yes	0 (0.0)	15 (57.5)	
No	16 (100.0)	11 (42.3)	
Attendant at delivery, n (%)			0.074(Fishers)
Traditional birth attendant/self	1 (8.3)	0 (0.0)	
Registered midwife	1 (8.3)	3 (20.8)	
Obstetrician	3 (16.7)	12 (54.2)	
Other medical practitioner	8 (66.7)	6 (25.0)	
Place of delivery, n (%)			0.847(Fishers)
Tertiary hospital	11 (68.2)	14 (66.7)	
Referral hospital	2 (12.5)	5 (23.8)	
General hospital	1 (5.6)	1 (4.8)	
Private hospital	1 (5.6)	1 (4.8)	
Home	1 (5.6)	0 (0.0)	
Place of death, n (%)			0.35
Intensive care unit	3 (18.8)	11 (42.3)	
Tertiary hospital	8 (50.0)	6 (23.1)	
Referral hospital	2 (12.5)	4 (15.4)	
General hospital	2 (12.5)	2 (7.7)	
Other	1 (6.2)	3 (11.5)	
**Pregnancy outcome**			
Days to death, n (%)			0.233(Fishers)
Pregnancy- 42 days post-partum			
Undelivered	6 (40.0)	4 (15.4)	
<24 hours	4 (26.7)	5 (19.2)	
1–6 Days	3 (20.0)	11 (42.3)	
7–42 Days	2 (13.3)	6 (23.1)	
Outcome of pregnancy, n (%)			0.862(Fishers)
Died undelivered	4 (26.7)	4 (15.4)	
Early fetal loss	2 (13.3)	3 (11.5)	
Stillbirth	2 (13.3)	4 (15.4)	
Live birth	7 (46.7)	15 (57.7)	
Survival of infant, n (%)			0.563
Yes	6 (37.5)	13 (50.0)	
No	8 (50.0)	12 (46.2)	
Not known	2 (12.5)	1 (3.8)	
Gestational age in weeks at termination of pregnancy/death (mean±SD)	31.5±7.5(n = 13)	32.9±8.0(n = 23)	0.627
Trimester pregnancy ended, n (%)			0.741(Fishers)
0–14 weeks	1 (6.7)	2 (7.7)	
15–28 weeks	4 (26.7)	4 (18.2)	
29+ weeks	10 (66.7)	20 (76.9)	
Birth Weight of live born infants, kgs(mean±SD)	2.31±0.60 (n = 5)	2.91±0.74(n = 9)	0.143
**Maternal mortality ratio**	**627.7**	**1119.7**	**1.78 (0.92**–**3.6)0.07**

*Calculated as Parity/ Gravidity: Defined as the proportion of pregnancies reaching viability.

**Figure 4:**
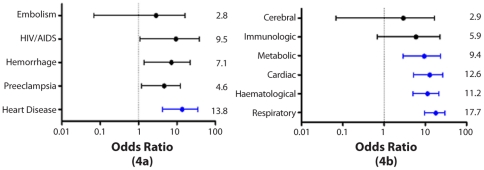
Odds Ratios for death in SCD by Underlying causes (4a) and Immediate causes (4b) of death.

Table 3 shows that estimated maternal mortality ratios (MMR) for women with SCD range between 551–719/100,000 compared to 78/100,000 among non SCD women, a relative ratio of 7 to 11 times. Even after excluding the forty-one percent of deaths (17/42) directly attributable to their sickling disorder, the relative risk remains 4 to 6 times higher than in the general population and represents the excess risk of dying in pregnancy attributed to SCD. When we examine closely the cause specific rates, in addition to their blood disorder, cardiovascular diseases were particularly challenging. Worth noting is that over the 10 year period no women with SCD died from endocrine disorders such as diabetes or thyrotoxicosis. They also experienced higher rates of hypertension and haemorrhage, the leading direct causes of death among non SCD women (Figure 4a). Rates of complications of treatment were much higher in the SCD population. The immediate cause of death among SCD women ran the gamut of organ failures, with respiratory failure, cardiac failure and haematological complications including coagulopathy carrying the highest odds, followed by hepato-renal failure (Figure 4b). Over the two time periods, the MMR increased, but not significantly (p value: 0.07), among SCD women from 628 to 1120/100 000 (Table 2).

**Table 3:** Cause specific underlying and immediate maternal mortality ratios per 100 000 live births, by sickle cell status, Jamaica, 1998–2007.

Underlying and immediate causes of death	Sickle cell maternal deaths		All other maternal deathsMMR (deaths)	Odds ratio (95% CI)	
	MMR (deaths)	Conservative estimate MMR			Conservative estimate
**Underlying cause of death**					
**TOTAL**	**718.8 (42)**	**551.4**	**77.8 (376)**	**11.1 (7.9 – 15.3)**	**7.1 (5.0**–**9.7)**
**Excluding blood disorders**	**427.9 (25)**	**328.2**	**77.6 (375)**	**6.6 (4.2**–**9.9)**	**4.2 (2.7**–**6.3)**
**Medical**	**555.2 (27)**	**354.5**	**24.4 (118)**	**22.7 (14.4**–**34.8)**	**14.4 (9.1**–**22.1)**
Blood disorders	349.6 (17)	223.2	0.2 (1)	1690 (265–70608)	1073 (168–44824)
Cardiovascular	102.8 (5)	65.6	7.4 (36)	13.8 (4.2–35.3)	8.8 (2.7–22.4)
HIV/AIDS	41.1 (2)	26.3	4.3 (21)	9.5 (1.1–38.8)	*6.0 (0.7*–*24.6)*
Respiratory	20.6 (1)	13.1	1.9 (9)	*11.1 (0.25*–*79.7)*	*7.0 (0.16*–*50.6)*
Endocrine	0		3.1 (15)	-	-
Other indirect	41.1 (2)	26.3	7.4 (36)	*5.5 (0.64*–*21.5)*	*3.5 (0.4*–*13.6)*
					
**Obstetric**	**246.8 (12)**	**157.5**	**47.4 (229)**	**5.2 (2.6**–**9.3)**	**3.3 (1.7**–**5.9)**
Hypertensive	82.3 (4)	52.5	17.8 (86)	4.6 (1.2–12.3)	2.9 (0.78–7.8)
Hemorrhagic	61.7 (3)	39.4	8.7 (42)	7.1 (1.4–22.2)	4.5 (0.89–14.1)
Embolism	20.6 (1)	13.1	7.4 (36)	*2.8 (0.07*–*16.4)*	*1.8 (0.04*–*10.4)*
Other direct	82.3 (4)	52.5	13.4 (65)	6.1 (1.6–16.4)	3.9 (1.0–10.4)
**Complications of treatment**	**61.7 (3)**	**39.4**	**6.0 (29)**	**10.3 (2.0**–**33.2)**	**6.5 (1.2**–**21.1)**
**Immediate cause of death (organ system failure)**					
Respiratory	329.0 (16)	210.1	18.6 (90)	17.7 (9.7–30.3)	11.2 (6.2–19.2)
Hematologic	205.6 (10)	131.3	18.4 (89)	11.2 (5.2–21.5)	7.1 (3.3–13.7)
Cardiac	164.5 (8)	105.0	13.0 (63)	12.6 (5.2–26.4)	8.0 (3.3–16.8)
Metabolic	102.8 (5)	65.6	11.0 (53)	9.4 (2.9–23.3)	6.0 (1.9–14.8)
Immunologic	41.1 (2)	26.3	7.0 (34)	*5.9 (0.68*–*22.8)*	*3.8 (0.4*–*14.5)*
Cerebral	20.6 (1)	13.1	7.2 (35)	*2.9 (0.07*–*16.9)*	*1.8 (0.04*–*10.7)*
No immediate cause stated	0		2.5 (12)	-	-
**Estimated births**	**4863** a	**7617** a	**483 387**		

Non significant differences in italics.

a Ministry of Health antenatal screening data, % of women screened for anaemia who have HbSS/SC disease, as a percent of all births: 2001–2007 (1.0%) (Table 3).

b Ministry of Health antenatal screening data, % of all first visits screened who have HbSS/SC disease, 2001–2007 (1.57%) – intention to treat, and applied to total registered live births to estimate births in SCD population (Table 3).

## Discussion

SCD remains a consistent cause of maternal mortality in Jamaica[15] and other populations of African and Mediterranean descent worldwide.[7], [19], [20], [21], [22] This is the largest series of maternal deaths in women with SCD able to quantify the relative difference in maternal mortality between women with SCD and the general population. After accounting for deaths directly attributed to their sickling disorder, the residual mortality ratio of 328–428/100,000 represents the excess risk that SCD poses to the childbearing population, a risk 4 to 6 times greater than in the general population. This excess risk underscores the need for urgent exploration of possible pathophysiological mechanisms to inform appropriate interventions. Notwithstanding, the interaction of abnormal rheology, leukocyte adhesion, endothelial dysfunction and inflammation of SCD with the physiological changes of pregnancy is of relevance.[23]

### Case Identification

Over the 10 year review period 42 maternal deaths among women with SCD were identified; 16 in the first five years, 26 in the latter. While increasing survival of women with SCD into the reproductive age group may explain this difference, it is also likely that with expansion in the high risk antenatal clinic network and better antenatal screening, more at risk women are being identified than previously. Limitations in sample size made it difficult to record statistical changes in clinical risks or outcomes over the two five year periods. We assumed it was unlikely for a woman with SCD to enter the reproductive age group without knowing she has SCD. Given the high recurrent cost of private care, these women would be regular users of public facilities and would either report their status or be identified during pregnancy. The more conservative estimate of the denominator for computing the MMRs uses their prevalence among first visits to public antenatal clinics (7617) or total births (4863).

While it is plausible that the clustering of cases in the south east region, where the Sickle Cell Unit is located, might suggest that families with affected persons may have relocated to this region to access these services, it is also possible that greater sensitization of the health team in this region has improved case identification, but further research is needed to explore this.

### Demographic characteristics

Consistent with data from Sergeant et al of reduced fecundity in this population, [7] our data indicate fewer conceptions as well. Over time it would appear that women were more willing to attempt to have a baby, and the average number of pregnancies increased between the first and second half of the study. Given their lower fecundity, women with SCD may be less interested in controlling their fertility and this might explain their relatively high prevalence in public antenatal clinics in excess of their expected distribution in the reproductive age group.

### Mortality risk

Bahrain reports a SCD prevalence similar to ours (0.9%)[24] but a significantly lower MMR of 25/100,000,[19] and attributes 25–30% of their maternal deaths to SCD[6], [19] compared to 10% in our setting. Rajab[25] suggests a MMR of 1140/100,000 (4 of 351 births) in their women with SCD (similar to 1120/100 000 in our second five year period). Without correcting for pregnancy outcomes (stillbirths and live births), this ratio is also similar to Serjeant's experience in Jamaica[4] (1220/100 000), which would be higher if the denominator was restricted to live births (1724/100 000).

The range of estimated maternal mortality among SCD women of between 551 and 719 compared to 78/100 000 in the general population demonstrates a 7 to 11 fold odds of dying in pregnancy. The upper estimate is consistent with prevalence data where 10% of deaths occurred among women with SCD who at birth made up less than 1% (1 in 150) of the population. The lower estimate is restricted to the population using public antenatal clinics. With an overall MMR of 87/100,000, it is not possible to reduce our maternal mortality ratio from 120 to 35 in keeping with the MDG5 goal, without improving the quality of care to this and other high risk populations.

### Access to obstetric referral care

In 1998 the Ministry of Health rolled out a national high risk antenatal care system[26], [27] to improve referral of women at risk of pregnancy complications for management by the obstetric teams at regional hospitals (see Figure 2). By 2002, 85% of antenatal women could access specialist care, although women had to travel beyond their usual community of residence. This increased access is reflected in the growth in SCD women using such services between 1998–2002 and 2003–07 (22% and 65% respectively).

Much of the emergency services required by women with SCD currently reside in tertiary facilities, far from where most women live. At minimum, ventilator support is needed in these facilities and will benefit other high risk women (e.g. hypertensive disorders, hemorrhage) who need short term access but for whom transfer to tertiary centres may not be efficient enough to save their lives and where there is already an increasing demand for ICU beds (with an ageing population, increasing trauma, etc.). The relative contribution of high risk women will only increase as more women delay childbearing into years when chronic diseases develop and as more women survive with these chronic disorders and attempt to reproduce.

### Quality of care

Most ‘delays’ [28] in care were due to patient factors or quality of care once the patient had been seen, with limitations in availability of blood, drugs and technical equipment. While ICU access improved ventilator support in the later period, these resources need to be better distributed as patients must be transferred to Kingston (south east) or Montego Bay (west). Common immediate causes of death were respiratory failure, coagulation defects and heart failure. Given the often acute and sudden deterioration in these patients, present efforts should be made to attend to their inpatient needs at tertiary facilities with ICUs.

A major challenge is ensuring that these women, their partners and families are educated about the risks to the mother and the genetic likelihood of passing the condition to their offspring. A comprehensive, integrated range of services should include family planning; preconception counselling to include partner screening and specialized antenatal care. The latter should include careful monitoring, education and counseling; appropriate dietary advice and supplementation; early identification of complications and problems; specialist medical care, appropriate pain control, physiotherapy and emergency obstetric care to ensure a successful and healthy pregnancy and delivery with minimum disease related complications.[29]–[30]

### Conclusion and recommendations

Access to high risk antenatal services and intensive care has improved in Jamaica but SCD continues to be a huge contributor to maternal mortality. Extensive education and counselling of women with SCD, their partners, and families contemplating pregnancy and during pregnancy is needed alongside better identification of SCD women early in pregnancy (comprehensive screening). Guidelines need to be created for all healthcare professionals to improve management of these women during pregnancy. Referral centres need reliable supplies of blood and blood products, ventilators, ability to screen for possible complications with use of e.g. echocardiography routinely during pregnancy, and must continue to closely follow-up of women postnatally.

## Supporting Information

Appendix S1 **Prevalence of sickle cell disease among users of public primary care antenatal clinics, Ministry of Health, Jamaica, 2003-7.**
(DOC)
