# Supplementary material for: Excess Risk of Maternal Death from Sickle Cell Disease in Jamaica: 1998–2007
Source: PLoS One. 2011 Oct 24;6(10):e26281. doi: 10.1371/journal.pone.0026281 (PMC3200316; doi:10.1371/journal.pone.0026281)
Supplement: Appendix S1 — Prevalence of sickle cell disease among users of public primary care antenatal clinics, Ministry of Health, Jamaica, 2003-7. (DOC) [file pone.0026281.s001.doc]

**Appendix 1: Prevalence of sickle cell disease among users of public primary care antenatal clinics, Ministry of Health, Jamaica, 2003-7**

| **Year** | **First visits to public antenatal clinics (% births)** | **Number of women screened for anaemia (%)** | **Number of women positive for HbSS·HbSC** | **% of all first visits** | **% of women screened for anaemia** | **% of all births** | **Registered live births** |
| --- | --- | --- | --- | --- | --- | --- | --- |
| 1998 | 35 712 (73·9) | 25 462 (71·3) | n/a |  |  |  | 48 139 |
| 1999 | 37 219 (70·9) | 27 395 (73·6) | n/a |  |  |  | 52 522 |
| 2000 | 37 929 (67·6) | 27 966 (73·7) | n/a |  |  |  | 56 134 |
| 2001 | 33 118 (66·9) | 26 531 (80·1) | 594 | 1·79 | 1·68 | 1·200 | 49 490 |
| 2002 | 32 034 (65·9) | 25 345 (79·1) | 391 | 1·22 | 1·54 | 0·804 | 48 627 |
| 2001-2 | 65 152 (66·4) | 51 876 (79·6) | 981 | 1·51 | 1·89 | 1·000 | 98 117 |
| 1998-2002 | 176 012 69·0) | 132 699 (75·4) |  |  |  |  | 254 912 |
| 2003 | 32 034 (68·0) | 23 545 (73·5) | 391 | 1·22 | 1·66 | 0·830 | 47 110 |
| 2004 | 30 037 (63·7) | 23 654 (78·7) | 444 | 1·47 | 1·88 | 0·942 | 47 127 |
| 2005 | 29 741(62·9) | 24 184 (81·3) | 533 | 1·79 | 1·83 | 1·128 | 47 254 |
| 2006 | 29 056 (62·8) | 23 447 (80·7) | 504 | 1·73 | 2·15 | 1·089 | 46 277 |
| 2007 | 26 843 (58·9) | 20 974 (78·1) | 450 | 1·68 | 2·15 | 0·987 | 45 590 |
| 2003-7 | 147 711(63·3) | 115 804 (78·4) | 2322 | 1·57 | 2·00 | 0·995 | 233 358 |
| 2001-7 | 212 863 (64·2) | 167 680 (78·8) | 3303 | 1·55 | 1·97 | 0·996 | 331 475 |
| 1998-2007 | 388 875 (66·3) | 300 379 (77·2) |  |  |  |  | 488 270 |
